# Effects of adding household water filters to Rwanda’s Community-Based Environmental Health Promotion Programme: a cluster-randomized controlled trial in Rwamagana district

**DOI:** 10.1038/s41545-022-00185-y

**Published:** 2022-09-12

**Authors:** Sabrina Haque, Miles A. Kirby, Laurien Iyakaremye, Alemayehu Gebremariam, Getachew Tessema, Evan Thomas, Howard H. Chang, Thomas Clasen

**Affiliations:** 1 Gangarosa Department of Environmental Health, Emory University Rollins School of Public Health, Atlanta, GA USA; 2 Department of Global Health and Population, Harvard T.H. Chan School of Public Health, Boston, MA USA; 3 Eagle Research Center, Kigali, Rwanda; 4 Catholic Relief Services, Kigali, Rwanda; 5 SNV-Rwanda, Kigali, Rwanda; 6 Mortenson Center in Global Engineering, University of Colorado, Boulder, CO USA

**Keywords:** Environmental social sciences, Development studies, Developing world, Environmental sciences

## Abstract

Unsafe drinking water remains a major cause of mortality and morbidity. While Rwanda’s Community-Based Environmental Health Promotion Programme (CBEHPP) promotes boiling and safe storage, previous research found these efforts to be ineffective in reducing fecal contamination of drinking water. We conducted a cluster randomized control led trial to determine if adding a household water filter with safe storage to the CBEHPP would improve drinking water quality and reduce child diarrhea. We enrolled 1,199 households with a pregnant person or child under 5 across 60 randomly selected villages in Rwamagana district. CBEHPP implementers distributed and promoted water purifiers to a random half of villages. We conducted two unannounced follow-up visits over 13–16 months after the intervention delivery. The intervention reduced the proportions of households with detectable *E. coli* in drinking water samples (primary outcome) by 20% (PR 0.80, 95% CI 0.74–0.87, *p* < 0.001) and with moderate and higher fecal contamination (≥10 CFU/100 mL) by 35% (PR 0.65, 95% CI 0.57–0.74*, p* < 0.001). The proportion of children under 5 experiencing diarrhea in the last week was reduced by 49% (aPR 0.51, 95%CI 0.35–0.73, *p* < 0.001). Our findings identify an effective intervention for improving water quality and child health that can be added to the CBEHPP.

## Introduction

Unsafe drinking water remains a leading risk factor for global mortality and morbidity, accounting for at least 1.23 million deaths and 65.1 million disability-adjusted life years (DALYs) from enteric infections in 2019^1^. While Rwanda achieved 82% coverage of access to improved water sources, a 22 percentage point increase since 2000^2,3^, three out of four Rwandan households rely on drinking water contaminated with fecal bacteria^4^. Enteric infections are currently the fifth leading cause of death of children under 5 in the country^5^, with unsafe drinking water contributing to an estimated 83% of diarrheal disease deaths in 2019^1^.

As countries work to develop reliable water supply systems, household water treatment and safe storage (HWTS) interventions (e.g., filtration, boiling, chemical disinfectants, solar disinfectant, use of covered collection and storage containers) serve as interim options for obtaining safe drinking water in the home. Various HWTS interventions have been shown effective to reduce diarrheal disease in settings with unsafe drinking water^6–8^. Though the disease burden from unsafe drinking water falls disproportionately on the poorest households living in low- and middle-income countries (LMICs), HWTS interventions seldom reach these populations at scale^9^. This implementation gap could be explained in part by a shortage of replicable, evidence-based models that work to achieve sustained coverage and use in disparate contexts^10–12^. The effectiveness of HWTS interventions on improving health depends on the acceptability and use of technologies in the population, pathogen environment, and the delivery and promotion strategy of the intervention, warranting a need for evidence-based models tailored to the local context^9,13^.

Rwanda has undertaken major initiatives for scaling up HWTS. In 2009, the Ministry of Health began the Community-Based Environmental Health Promotion Programme (CBEHPP) as its primary strategy to combat childhood diarrheal disease. CBEHPP adapts a “Community Health/Hygiene Club” (CHC) approach to promote hygienic practices, intending to achieve zero open defecation, at least 80% hygienic latrine coverage, and improvements in water handling as well as handwashing. The program operates throughout Rwanda, with nearly all villages forming a CHC and implemented by the Ministry of Health working through local authorities and a consortium of NGOs and international donors^14^. While the CBEHPP primarily promotes boiling with safe storage, only 10–34% of Rwandan households report the practice^4,15^. Moreover, a 12-month trial of the CBEHPP in Rwanda’s Rusizi district found that the program did not improve drinking water quality or reduce diarrhea or nutritional outcomes in young children, even with increases in reported boiling and other safe water handling and treatment practices and access to improved sanitation facilities^16^. The authors speculate that CBEHPP is likely ineffective in improving health because it overly relies on hygiene behavior promotion without the provision of effective WASH hardware to enable households to act on acquired knowledge.

The 2014 *Tubeho Neza* (“Live well” in Kinyarwanda) campaign is another significant effort to scale HWTS in Rwanda. The social enterprise DelAgua Health, in cooperation with the Ministry of Health, delivered tabletop *Lifestraw Family 2.0* purifiers and improved cookstoves free of cost to over 100,000 households belonging to the lowest economic quartile in Rwanda’s Western Province^17^. The campaign involved an intensive effort to promote full coverage and correct and exclusive use of the filter in the target population. Promotional activities included community education (e.g. meetings, skits, radio advertisements), behavior-change materials, and regular household visits by community health workers (CHWs) paid by the implementer to repair or replace failed units, address issues, and reinforce the need for consistent use of the filter by all household members^17^. Kirby and Nagel et al.^18^ found that the intervention reduced the proportion of households with detectable fecal contamination in drinking water samples by 38% (PR 0.62, 95% CI 0.57–0.68) and caretaker-reported child diarrhea by 29% (PR 0.71, 95% CI 0.59–0.87) over 12 months. Lower seroprevalence of immunoglobulin G (IgG) antibody response to *Cryptosporidium* was also observed in intervention children under 2 (RR 0.62, 95% CI 0.44–0.89)^19^. Although an effective intervention and one of the largest distribution of these filters to date, *Tubeho Neza* is no longer operating and has not been replicated in other regions of Rwanda due to inadequate financing to continue the intensive campaign and household-level support that characterized the *Tubeho Neza* initiative.

Western Province’s filter campaign was an effective model for improving point-of-use water quality and child health outcomes in a large vulnerable population in Rwanda. Separately, the CBEHPP succeeds in mobilizing WASH actors and establishing sustained village institutions dedicated to promoting hygienic behaviors nationally; however, it does not effectively improve access to safe water nor reduce childhood diarrhea. The Government of Rwanda is considering ways to strengthen CBEHPP implementation. One option is integrating components of the evidence-based model of delivering household filters as similar to the *Tubeho Neza* campaign into CBEHPP. CBEHPP’s existing institutional infrastructure could be leveraged as a platform to scale promotion and delivery of the filter. It is uncertain, however, whether the technology can achieve similar results when provided with the less resource-intensive approach that differentiates the CBEHPP’s CHC model from the *Tubeho Neza* program.

This study was designed to address whether filters can be delivered as part of the CBEHPP in a manner that improves household drinking water quality. We hypothesized that the intervention would improve drinking water quality as measured by the proportion of samples with detectable *E. coli* and contamination levels at moderate or higher risk (≥10 CFU/100 ml) and very high risk (≥100 CFU/100 mL). We report effects on the primary outcome on *E. coli* presence in drinking water and secondary outcomes on coverage and uptake of the filter, caregiver-reported diarrhea among children under 5, and reported healthcare visits for diarrhea treatment among children under 5 over 13–16 months.

## Results

### Study participants

**Fig. 1:**
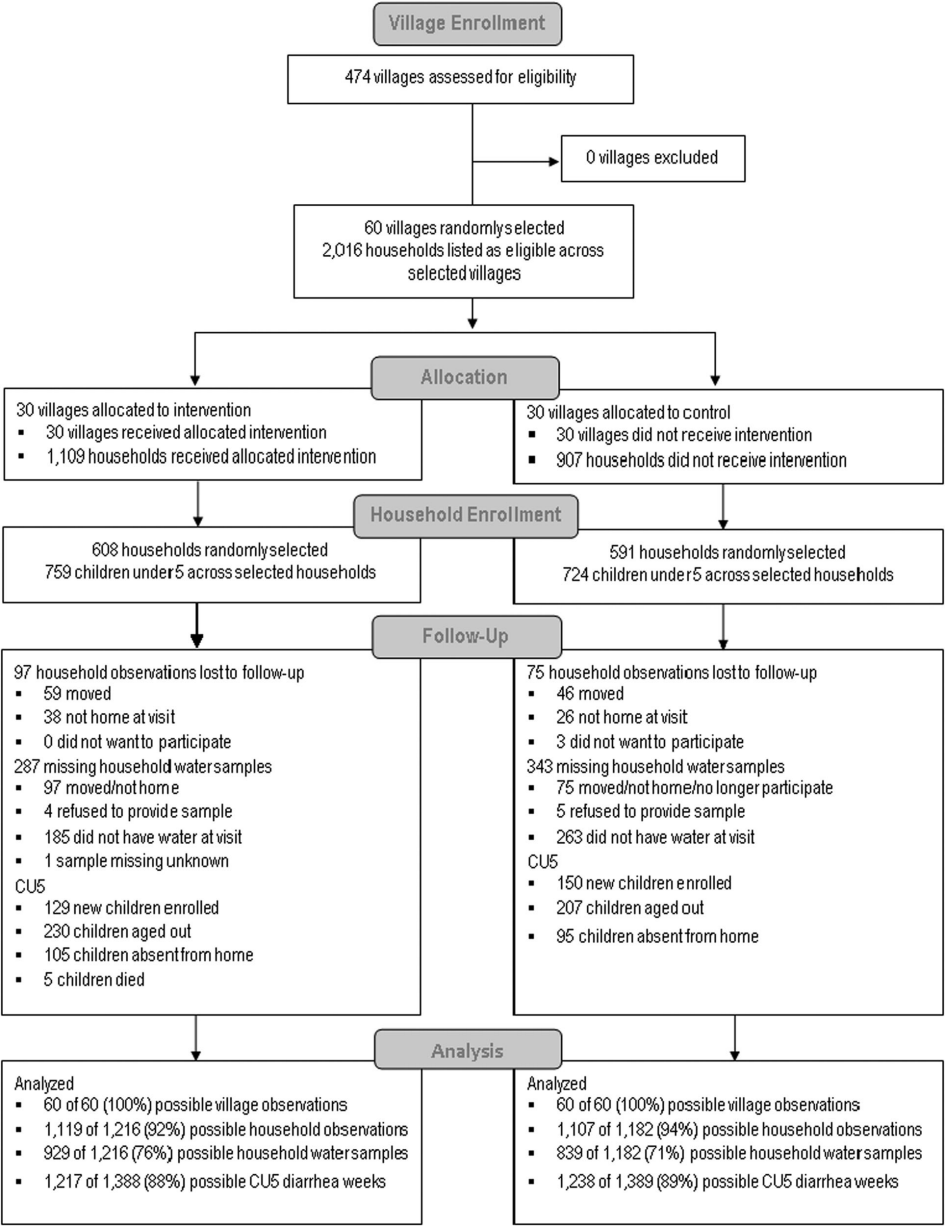
Trial flow diagram. CONSORT flow diagram of enrollment and follow-up of study participants.

1,109 households across intervention villages and 907 households across control villages were identified as eligible according to the inclusion criteria. For the evaluation, 608 and 591 households were randomly selected in the intervention and control groups, respectively. All households selected to be enrolled into the study provided written consent to participate. At baseline, 759 and 724 children under 5 years of age were enrolled into the intervention and control groups, respectively (Fig. 1). We enrolled an average of 20 households per village (SD: 5; range: 10–36 households). Enrollment in seven villages exceeded our cap of 25 households (26 households in five villages and 29 and 36 households in two villages) due to communication barriers in the field.

Baseline characteristics by study group are reported in Table 1. Access to a place for handwashing, access to improved sanitation, and government-defined socio-economic status had appreciable differences between study groups and were examined as potential confounders in separate sensitivity analyses of adjusted models of the effects. The adjusted model with government-defined socio-economic status made a three percentage-point difference on the effect on diarrhea.

**Table 1:** Household and child characteristics at baseline by study arm.

Characteristic	Intervention Group	Control Group
Demographic and household information		
Number of households	608	591
Percent female respondent	92.8% (564/608)	93.4% (550/589)
Percent female household head	7.1% (43/606)	7.1% (42/589)
Percent respondents completed primary school or higher	50.9% (308/605)	49.8% (293/588)
Percent household head completed primary school or higher	51.5% (296/575)	43.3% (244/563)
Percent household belongs to *Ubudehe* I or II (lowest government-defined socio-economic classes)	48.8% (294/603)	34.5% (202/586)
Percent household-owned house	89.4% (539/603)	89.0% (525/590)
Percent household had electricity	59.1% (359/607)	57.8% (341/590)
Percent household-owned livestock	61.7% (375/608)	66.8% (395/591)
Percent household floor material made of earth/sand	70.9% (431/608)	71.4% (422/591)
Mean respondent age in years (SD)	34.6 (9.9)	34.3 (9.8)
Mean household head age in years (SD)	40.6 (12.1)	41.0 (12.1)
Mean number of residents in household (SD)	5.1 (1.7)	5.1 (1.7)
Mean number of rooms (SD)	5.1 (1.8)	5.2 (1.9)
Sanitation and hygiene		
Percent access to JMP improved sanitation	72.0% (437/607)	80.0% (473/591)
Percent evidence of chickens or cows in compound or yard	29.0% (176/608)	29.8% (176/591)
Percent has a handwashing location	37.4% (227/607)	42.3% (250/591)
Drinking water source and practices		
Percent main drinking water source: JMP Improved	89.1% (542/608)	86.6% (512/591)
Percent main drinking water source: JMP Basic Water (Improved < 30 min. roundtrip)	25.5% (155/608)	26.7% (158/591)
Percent piped water to dwelling or yard/plot	12.3% (75/608)	13.2% (78/591)
Percent piped water to neighbor	6.4% (39/608)	9.3% (55/591)
Percent public tap/stand pipe	32.7% (199/608)	27.2% (161/591)
Percent protected spring or well	37.0% (225/608)	36.5% (216/591)
Percent unprotected spring or well	2.6% (16/608)	3.2% (19/591)
Percent surface water	5.4% (33/608)	6.9% (41/591)
Percent no reported drinking water treatment practice	48.3% (293/606)	52.7% (311/590)
Percent observed to store drinking water	95.4% (395/414)	94.5% (415/439)
Drinking water quality (point-of-use)		
Percent <2 CFU/100 mL (no detectable *E. coli*)	9.4% (39/414)	4.6% (20/438)
Percent 1–10 CFU/100 mL	16.4% (68/414)	15.3% (67/438)
Mean *E. coli* CFU/100 mL	207.4 (272.1)	215.4 (298.3)
Child Under 5 Years of Age Characteristics		
Percent female	44.8% (340/759)	47.7% (345/724)
Percent with caretaker-reported 7-day diarrhoea	6.5% (49/752)	7.3% (52/712)
Percent with completed rotavirus vaccination (3-dose series observed on vaccination card	80.2% (412/514)	80.9% (390/482)
Mean age in months (SD)	30.4 (16.6)	30.0 (16.3)

A total of 2226 household observations and 2455 child observations were analyzed at midline and endline visits, respectively. Attrition of observations was slightly higher in the intervention group (Fig. 1). Reasons for lost to follow up include moving away, unavailable at time of visit (e.g., enumerators visited household at least twice in a day, with at least 2-h between visits), or households no longer wished to participate. Five children died in the intervention arm. Deaths were reported to the Emory IRB and RNEC, but deemed unrelated to the intervention. We found no evidence that attrition was dependent on characteristics of the household or respondent, such as socio-economic status, education level of the respondent, access to improved water sources, and roundtrip time to collect water.

### Filter Coverage, Use, and Acceptability

Table 2 provides data on filter coverage, use, and acceptability at midline and endline visits in the intervention group. In combined data from both follow-ups, the filter was observed to be in 99% and functioning in 93% of intervention households. About 95% of intervention households reported using the filter, with 97% at midline and 94% at endline. There was a decline in the percentage of households reporting filling the filter in the previous 7 days, from 97% in midline to 92% in endline. We also found a decline in filters that were observed to have water in the storage container from 81% in midline to 75% in endline. Overall, 81% of households with children under the age of 5 reported that at least one child drank filtered water the previous day. Fewer households reported to treat the provided water sample with the filter at endline, dropping from 95% to 81%. Overall, households generally accepted the filter in terms of water appearance, water smell, water taste, and time to filter water. The amount of time to treat water was the least acceptable feature of the filter.

**Table 2:** Coverage, use, and acceptability of filter at midline and endline in intervention group.

	Midline		Endline		Overall	
Coverage	*N*	% (95% CI)	*N*	% (95% CI)	*N*	% (95% CI)
Filter observed in household	555	99.1 (97.9, 99.6)	563	97.9 (96.3, 98.8)	1,118	98.5 (97.6, 99.1)
Filter observed to be in good condition^a^	532	94.0 (91.6, 95.7)	507	91.9 (89.2, 94.0)	1,039	93.0 (91.2, 94.4)
Use						
Filter reported to be used currently	552	96.6 (94.7, 97.8)	551	93.6 (91.3, 95.4)	1,103	95.1 (93.7, 96.2)
Filter reported to be filled in last 7 days	543	96.9 (95.0, 98.0)	550	91.6 (89.0, 93.7)	1,093	94.2 (92.7, 95.5)
Storage container of filter observed to have water in it	544	81.4 (77.9, 84.5)	545	75.0 (71.2, 78.5)	1,089	78.2 (75.7, 80.6)
Drinking water sample provided reported to be treated by Lifestraw filter	447	94.6 (92.1, 96.4)	482	80.7 (76.9, 84.0)	929	87.4 (85.1, 89.4)
Report at least one young child drank filtered water yesterday	535	83.7 (80.4, 86.6)	523	78.8 (75.1, 82.1)	1,058	81.3 (78.8, 83.5)
Acceptability^b^						
Appearance of filtered water	549	100	551	100	1,100	100
Smell of filtered water	550	99.1 (97.8, 99.6)	551	99.5 (98.3, 99.8)	1,101	99.3 (98.6, 99.6)
Taste of filtered water	548	99.5 (98.3, 99.8)	546	99.6 (98.5, 99.9)	1,094	99.5 (98.9, 99.8)
Time to filter water	549	91.4 (88.9, 93.5)	551	88.2 (85.2, 90.6)	1,100	89.8 (87.9, 91.5)

N denotes the number of household observations in survey round.

^a^Good condition refers to being observed to have been assembled properly, working tap, no leaking, undamaged container, adequate flowrate, and ability to backwash.

^b^Respondent reported feature to be acceptable or very acceptable.

### Drinking water quality

A total of 929 and 839 water samples were collected during follow-ups in the intervention and control groups, respectively. The control group had more missing water samples compared to the intervention group. Reasons for missing water samples were either due to household lost to follow up, lost sample, or more commonly because households did not have available drinking water at the time of visit (Fig. 1). We did not find evidence that missing water samples in the overall study population were statistically related to observed household or respondent characteristics plausibly related to drinking water availability. In a stratified analysis of the study arms, increasing household size was associated with higher prevalence of obtaining a water sample in the intervention group (PR 1.02, 95% CI 1.00–1.03 *p* value= 0.02). However, household size was balanced between arms, so there is likely limited risk of bias due to missingness and household size.

**Fig. 2:**
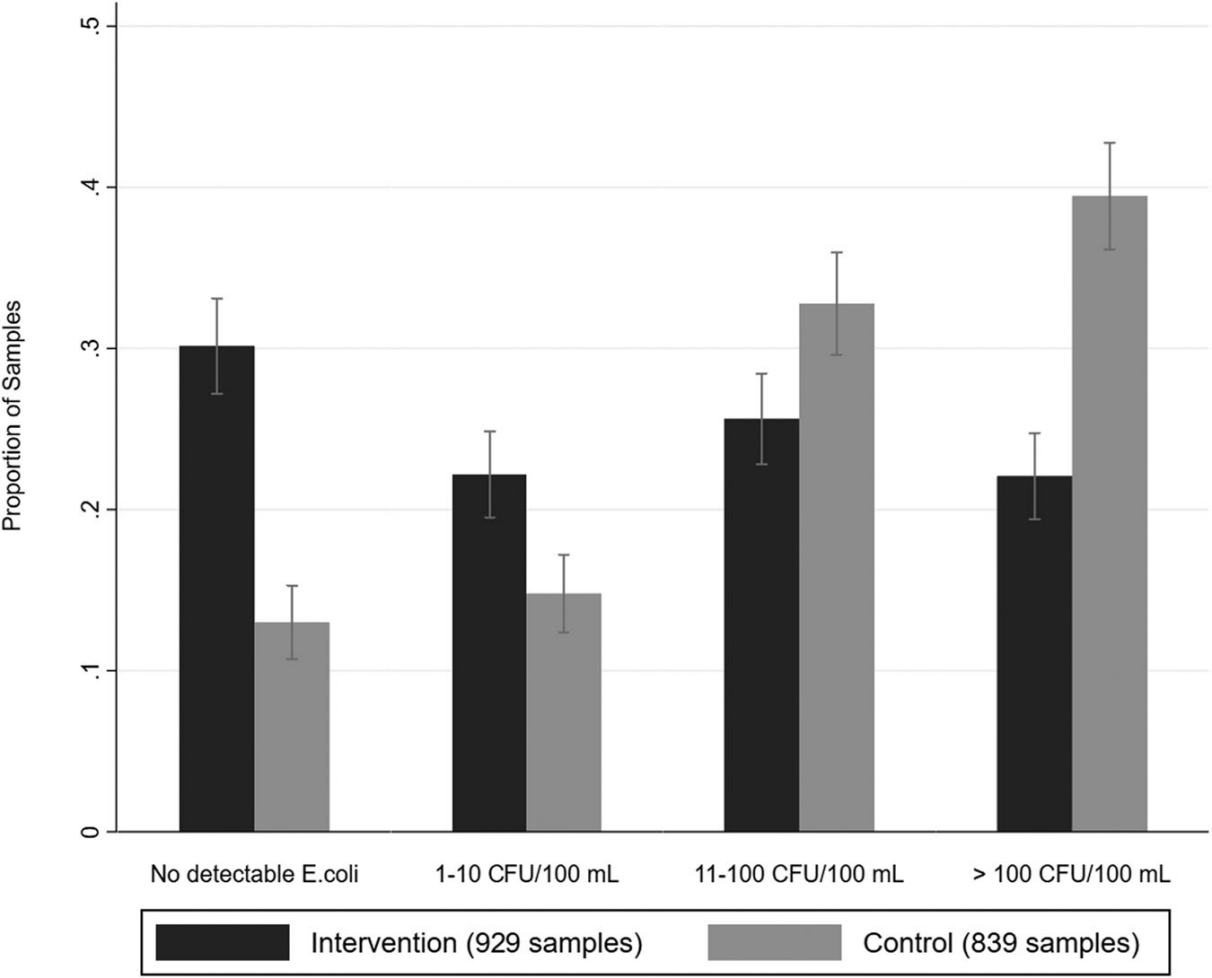
Distribution of water quality result by WHO risk level (midline and endline combined). Bar graphs of the proportions of point-of-use water samples over the follow-up period that presented no detectable *E. coli*, 1–10 *E. coli* colony forming units (CFU), 11–100 *E. coli* CFU, or >100 *E. coli* CFU per 100 milliliters of water. Bar graphs stratified by study groups. Error bars represent 95% confidence intervals.

Overall, the proportion of drinking water samples with no detectable *E. coli* was higher in the intervention group (Fig. 2). Table 3 shows the effects of the intervention on the drinking water quality, analyzed by detectable *E. coli* and other WHO risk categories^20^. Prevalence ratios (PR), 95% confidence intervals (95% CI) and *p* values are derived from log-binomial generalized estimating equations with robust standard errors to account for clustering within villages. The intervention reduced the proportion of drinking water with detectable *E. coli* by 20% (PR 0.80, 95% CI, 0.74–0.87, *p* value < 0.001) compared to the control. It reduced the proportion of drinking water samples with moderate and higher fecal contamination (≥10 CFU/100 mL) by 35% (PR 0.65, 95% CI 0.57–0.74, *p* value < 0.001) and the proportion of drinking water samples with very high fecal contamination (≥100 CFU/100 mL) by 44% (PR 0.56, 95% CI 0.46–0.69, *p* value < 0.001). The adjusted models did not differ with crude models of effects on water quality outcomes. The improvement in drinking water quality among intervention households is also evident in comparing mean levels of colony-forming units (CFUs) of *E. coli* (Supplementary Table 1). The intervention group had an arithmetic mean of 91.8 CFU/100 mL (95% CI 80.6–103.1) and Williams mean of 14.1 CFU/100 mL (95% CI 12.3–16.1), and the control group had an arithmetic mean of 175.3 CFU/100 mL (95% CI 158.3–192.2) and Williams mean of 44.4 CFU/100 mL (95% CI 38.8–50.8).

**Table 3:** Effects of intervention during follow-up on household-level drinking water quality outcomes.

Model	Drinking water quality	Intervention	Control	PR (95% CI)	*p*
1^a^	≥2 CFU/100 mL (any detectable *E. coli* contamination)	69.9% (649/929)	87.0% (730/839)	0.80 (0.74, 0.87)	<0.001
2^a^	≥10 CFU/100 mL (Moderate and higher *E. coli* contamination)	49.3% (458/929)	74.7% (627/839)	0.66 (0.58, 0.75)	<0.001
3^a^	≥100 CFU /100 mL (Very high *E. coli* contamination)	22.4% (208/929)	39.8% (334/839)	0.56 (0.46, 0.68)	<0.001
4^b^	≥2 CFU/100 mL (any detectable *E. coli* contamination)	69.8% (644/923)	87.2% (728/835)	0.80 (0.74, 0.87)	<0.001
5^b^	≥10 CFU/100 mL (Moderate and higher *E. coli* contamination)	49.2% (454/923)	75.0% (626/835)	0.65 (0.57, 0.74)	<0.001
6^b^	≥100 CFU /100 mL (Very high *E. coli* contamination)	22.4% (207/923)	39.9% (333/835)	0.56 (0.46, 0.68)	<0.001

n denotes the total number of household water samples analyzed in follow-up rounds.

^a^Prevalence ratio (PR), 95% Confidence Interval (95% CI) and *p* value derived from log-binomial generalized estimating equations with robust standard errors to account for clustering within village. Model only conditions group assignment and drinking water quality outcome.

^b^PR, 95% CI and *p* value derived from log-binomial generalized estimating equations with robust standard errors to account for clustering within village. Model further adjusts for government-defined socioeconomic status.

### Child Diarrhea

The intervention reduced the prevalence of child diarrhea (Table 4). PRs, 95% CIs and *p* values are derived from log-binomial generalized estimating equations with robust standard errors to account for clustering within villages. Among children under 5, diarrheal prevalence in the previous 7-days was reduced by 49% (PR 0.51, 95% CI 0.35–0.73, *p* value < 0.001) after adjusting for government-defined socioeconomic status. Similar effects were seen in children under two (PR 0.55 95% CI 0.37–0.83, *p* value = 0.005). Caregivers reported fewer visits to CHWs or clinics for diarrhea treatment for children under 5 (PR 0.46, 95% CI 0.22–0.96, *p* value = 0.039) after adjusting for government-defined socioeconomic status. Although the proportion of reported visits to CHC or clinics was lower in the intervention group among children under two, there were no overall effects from the intervention on CHW or clinic visits for this age group. The effects on child diarrheal outcomes observed from the models unadjusted for socioeconomic status are slightly lower compared to adjusted models. As the trial was unblinded and the health outcomes were reported, we used reported toothache as a negative control to explore possible reporting bias^21^. Effects were borderline significant in the unadjusted model, but in the opposite expected direction (PR 1.58, 95% CI 1.00–2.52, *p* value = 0.052); they were not statistically significant when adjusted for SES imbalance (aPR 1.55, 95% CI 0.95–2.52, *p* value = 0.290).

**Table 4:** Effects of intervention during follow-up on diarrhea outcomes for children under 5 and 2.

Model	Secondary Outcome	Intervention	Control	PR (95% CI)	*p*
	Diarrhea- Children Under 5				
1^a^	In the last 7 days, child reported to have 3 or more loose stools in 24 h	4.8% (59/1,217)	9.3% (115/1,238)	0.54 (0.38, 0.78)	0.001
2^a^	In the last 7 days, child reported to be taken to CHW or clinic for diarrhea treatment	1.5% (18/1,222)	3.0% (37/1,243)	0.53 (0.28, 0.98)	0.045
3^a^	In the last 7 days, child reported to be taken to CHW for diarrhea treatment	0.6% (7/1,223)	1.2% (15/1,243)	0.48 (0.18, 1.26)	0.138
4^a^	In the last 7 days, child reported to be taken to clinic for diarrhea treatment	1.0% (12/1,222)	2.1% (26/1,243)	0.48 (0.23, 0.98)	0.043
	Diarrhea- Children Under 2				
5^a^	In the last 7 days, child reported to have 3 or more loose stools in 24 h	8.7% (33/379)	15.6% (58/371)	0.55 (0.37, 0.83)	0.005
6^a^	In the last 7 days, child reported to be taken to CHW or clinic for diarrhea treatment	3.2% (12/379)	5.7% (21/370)	0.52 (0.26, 1.02)	0.059
7^a^	In the last 7 days, child reported to be taken to CHW for diarrhea treatment	0.8% (3/379)	1.9% (7/370)	0.45 (0.11, 1.85)	0.268
8^a^	In the last 7 days, child reported to be taken to clinic for diarrhea treatment	2.6% (10/379)	3.8% (14/370)	0.63 (0.29, 1.39)	0.255
	Diarrhea- Children Under 5				
9^b^	In the last 7 days, child reported to have 3 or more loose stools in 24 h	4.9% (59/1,206)	9.3% (115/1,233)	0.51 (0.35, 0.73)	<0.001
10^b^	In the last 7 days, child reported to be taken to CHW or clinic for diarrhea treatment	1.5% (18/1,211)	3.0% (37/1,238)	0.52 (0.27, 0.98)	0.044
11^b^	In the last 7 days, child reported to be taken to CHW for diarrhea treatment	0.6% (7/1,212)	1.2% (15/1,238)	0.47 (0.18, 1.27)	0.138
12^b^	In the last 7 days, child reported to be taken to clinic for diarrhea treatment	1.0% (12/1,211)	2.1% (26/1,238)	0.46 (0.22, 0.96)	0.039
	Diarrhea- Children Under 2				
13^b^	In the last 7 days, child reported to have 3 or more loose stools in 24 h	8.8% (33/377)	15.6% (58/371)	0.55 (0.37, 0.83)	0.005
14^b^	In the last 7 days, child reported to be taken to CHW or clinic for diarrhea treatment	3.2% (12/377)	5.7% (21/370)	0.51 (0.25, 1.02)	0.057
15^b^	In the last 7 days, child reported to be taken to CHW for diarrhea treatment	0.8% (3/377)	1.9% (7/370)	0.49 (0.12, 2.08)	0.334
16^b^	In the last 7 days, child reported to be taken to clinic for diarrhea treatment	2.7% (10/377)	3.8% (14/370)	0.61 (0.27, 1.37)	0.229

n* denotes the total number of child observations analyzed in follow-up rounds.

^a^Prevalence ratio (PR), 95% Confidence Interval (95% CI) and *p* value derived from log-binomial generalized estimating equations with robust standard errors to account for clustering within village. Model conditions group assignment, age in months, sex, and diarrhoea outcome.

^b^PR, 95% CI and p-value derived from log-binomial generalized estimating equations with robust standard errors to account for clustering within village. Model further adjusts for government-defined socio-economic status.

## Discussion

Our results show that adding filter delivery to the CBEHPP in Rwamagana district improved drinking water quality and reduced diarrheal prevalence and reported CHW visits for diarrhea in the previous 7-days among children under 5. Thirteen to 16 months after delivery, we observed nearly universal coverage of filters in households and found that the intervention increased household-reported treatment of drinking water. While delivery and support of the filter was less intensive than the *Tubeho Neza* program in Western Province, the results show the intervention to be similarly protective in the CBEHPP^18^. These findings are in contrast to previous evidence showing no improvement in water quality or diarrhea under current approaches to the CBEHPP that depend only on behavior-change communication^16^.

Nevertheless, we saw evidence of decreasing trends in filter condition, use, and acceptability between rounds. For example, the proportion of households providing drinking water samples reportedly treated by the filter dropped by 14 percentage points from the first follow-up visit. Households also reported less acceptability of the duration it took filter water by the end of the follow-up. The declining trends in the intermediate outcomes could be the result of seasonality effects on water handling practices in follow-up visits, unsustainable behavior-change, or filter breakage over time^21,22^. In our study population, we observed that the proportion of households using unimproved water sources for their primary drinking water source nearly doubled in the dry season (i.e., endline) in both the intervention and control groups. The use of highly turbid water may influence filter condition, such as risk of clogging and requiring more time for water to pass through the purification system^23^. In parallel to scaling HWTS interventions, governments should invest in long-term improvements to water supply to fully realize health goals.

Program slippage overall is also common in WASH programs, where households gradually resort back to original practices pre-intervention. Notably, most protective effects from HWTS interventions on drinking water quality and health are among studies with short follow-up periods (e.g., <12 months)^6^. Studies that have done follow-up work on HWTS evaluations have been mixed in showing sustained impact, but overall, most show that there is significant decline in use^18,22,24–26^. The positive health effects observed in shorter duration trials could also reflect attenuation in implementation intensity, where the effectiveness of HWTS interventions on health is likely dependent on the frequency of contact between behavior-change promoters and households^27^. In our context, we note that there was more implementation activity, including one planned filter promotional and maintenance visit to households, in the first 6 months of follow-up in the intervention group. Additionally, COVID-19 restrictions and sporadic lockdowns beginning in March 2020 may have constrained the ability to regularly hold and attend CHC meetings in the latter portion of the study period among both groups. The observed decline in use in the intervention group supports a need for more deliberate implementation efforts to upkeep use and functionality of HWTS innovations.

Although our study is limited to 13–16 months of follow-up, it is one of the few long-term evaluations showing positive effects on drinking water and health using an HWTS implementation model. In a matched cohort study, Kirby and colleagues^26^ showed that water quality effects from similar filters in Rwanda can be sustained for over 2 years if replace and repair mechanisms are in place. Regardless of declining trends in intermediate outcomes, we found that drinking water quality and child diarrheal prevalence was consistently better compared to the control group at both midline and endline visits. Additional follow-up rounds at the conclusion of the trial were planned over the next 12 months to further evaluate trends in implementation activities, coverage, use, acceptability, functionality, drinking water quality, and diarrhea.

Our study has limitations including the nonblinded nature of the intervention and reliance on reported outcomes, presenting the risk of courtesy/social desirability and recall bias on reported use and health outcomes. Observed water in the filter may not indicate consistent use^28^, and unannounced visits are still vulnerable to household reactivity bias on observational outcomes^29^. Nevertheless, the positive effects on use and diarrheal disease prevalence are reinforced through reductions in *E. coli* contamination in drinking water samples and null effects on health outcomes unrelated to intervention such as 7-day prevalence of toothaches. Although cross-sectional measurement of household drinking water quality is an imperfect proxy for exposure to fecal-contaminated water and disease risk in the preceding week of the survey^30–32^, it is demonstrated to significantly increase the risk of waterborne illnesses^33,34^. The dependence on reported diarrhea also does not capture sub-clinical infections related to contaminated drinking water and other exposures^35^.

Our evaluation supports the intervention’s underlying theory of change that the delivery and promotion of locally-acceptable HTWS hardware improves drinking water quality and, subsequently, child health. Our findings suggest that Rwanda’s CBEHPP and other similar programs using CHC models can be used to deliver acceptable HWTS technologies to vulnerable communities. The integration of microbiologically proven filters into the CBEHPP is one evidence-based option that may help the program meet its objectives of reducing the diarrheal burden in Rwanda. Future studies should document the long-term sustainability and use of HWTS hardware, given the trends in declining use and condition observed over 13–16 months. Research should also examine strategies for ongoing monitoring (e.g., use and drinking water quality) and compare and optimize implementation strategies that will help policymakers and development partners feasibly scale safe drinking water solutions in their local contexts.

## Methods

We conducted a cluster randomized controlled trial in Rwamagana district to determine whether adding a household-based water filter with safe storage to the CBEHPP could be effective in improving drinking water quality.

### Intervention

The intervention under evaluation is the delivery and promotion of the LifeStraw Family 2.0 filters in the CBEHPP program. The filter is a tabletop point-of-use water treatment system that includes an 80 μm pre-filter to remove coarse material, 20 nm hollow-fiber ultrafiltration membrane, backwash lever, and covered storage container with 5.5 L capacity. The system meets the WHO’s “comprehensive protection” guideline for household water treatment technologies^36^; it can filter up to 18,000 liters of water, which should be able to supply a family of five with clean drinking water for three to five years, without any replacement of parts^37^.

Delivery and promotion of the filter is through the CBEHPP, which organizes village-level CHCs with a maximum membership of one hundred households. Clubs aim to meet weekly and are led by volunteer CHC facilitators that are trained to deliver a 20-module curriculum designed by the Ministry of Health. The filter-integrated intervention tasks CHC facilitators to additionally serve as the primary service providers of the filter. The CBEHPP filter integration was intended to have “lighter touch” engagement compared to the delivery of filters in the *Tubeho Neza* campaign. Major differences between the approaches include *Tubeho Neza’s* additional delivery of improved cookstoves, exclusive targeting of households belonging to the lowest economic quartile, mass media campaigns, and supplementary promotional activities such as regular CHW cooperative and community meetings and frequent household visits (Barstow et al. 2016).

Bradshaw et al.^38^ publish further details on the intervention and delivery in their process evaluation. CHC facilitators were trained to promote the filter and to repair or replace nonfunctional units. Eligible households were invited to receive the filter at a mass-distribution event held at the main health center serving the geographical sector. Following the distribution, CHC facilitators conducted individual household visits to teach households how to use the filter and provide a promotional poster. Households were instructed not to use the filter until the initial visit was completed. A second promotional household visit by CHC facilitators was completed ~6 months later to monitor upkeep/functionality, use, and satisfaction with the filter. CHC facilitators additionally reinforced messaging in CHC meetings. Households that were eligible to receive the filter included CHC members and had at least one child under the age of 5 or had at least one pregnant woman living in the household. All eligible households were able to receive the filter regardless of being selected to participate in the study.

Catholic Relief Services (CRS) and SNV, two of the government’s primary implementing NGO partners of CBEHPP, delivered the intervention with their local partner African Evangelistic Enterprise (AEE). The NGOs implement CBEHPP and its CHC model through *Gikuriro*, a USAID WASH and nutrition program. SNV, CRS, and AEE were supported in this initial distribution and promotion by Amazi Yego, the social enterprise that collaborated in the *Tubeho Neza* filter promotion in Western Province^17^. Amazi Yego trained CRS, SNV, and AEE and shared experiences in filter delivery. Amazi Yego was also significantly involved in designing the implementation protocol, providing promotional material to be provided to householders and implementing the intervention alongside CRS/SNV/AEE.

### Study design

We employed a cluster-randomized controlled trial design to assess the effects of the intervention on point-of-use (POU) drinking water quality as the primary outcome; we also assess intervention coverage and use and effects on reported diarrhea as secondary outcomes. The trial was conducted over 13–16 months in two follow-up visits. Rwamagana is a primarily rural district in Rwanda’s Eastern Province and has a population of 313,461 people^39^. Rwamagana was selected because it is located in Eastern Province, which has one of the highest rates of fecal contamination of drinking water in the country^4^ and because it was one of the districts the implementers worked in. SNV and CRS are active in all 474 villages across Rwamagana.

Sixty villages (clusters) were randomly selected, with 30 receiving the intervention (CBEHPP + filter) and 30 serving as controls (CBEHPP alone). Villages were randomly selected from a list of the 474 eligible villages using probability proportional-to-size sampling (PPS) without replacement using *samplepps* in Stata 16 software^40^. PPS was done based on the implementer’s reported size of the CHC in each village.

Households in selected villages were eligible to participate in the study if they were verified eligible to receive the intervention (CHC member households who had at least one child under 5 or pregnant person living in the household at time of baseline) and had a household member that was over 18 years of age available to consent to enrollment. A list of eligible households was made for each of the 60 villages by consulting the district registers, CHC registers, and with the CHC facilitators. Eligible households per village ranged from 10–72 households. Twenty-five households were randomly selected to be enrolled in the study from each village list using simple random sampling using the sample function to randomly order households in *R* statistical software^41^. Other eligible households were deemed as replacement study households. Enumerators were instructed to attempt each of the randomly selected 25 households twice at least 2 h apart during the day. If households could not be reached or were otherwise found to be ineligible, enumerators enrolled one of the replacement households based on a random order. To complete a village, at least half of the eligible households in the village needed to have been enrolled, but a cap of 25 households per village was enforced due to logistical constraints.

### Randomization and blinding

Random allocation of the intervention and control groups was done at the village level. To help ensure geographical balance between arms, random allocation of the intervention was stratified by the 13 sectors within the district. An individual unaffiliated with the project conducted the allocation. The data collection team, village-level implementers/leaders (e.g., CHC facilitators, village leaders, CHWs, AEE staff) and participating households were blinded to the allocation during baseline data collection. Enumerators and households could not be blinded after implementation due to the nature of the intervention. The primary data analyst additionally oversaw and managed the data collection, and therefore, could not be blinded. The principal investigator remained blinded throughout the study duration.

### Baseline and follow-ups

A baseline survey was conducted from December 2018 to March 2019 prior to intervention delivery. The intervention was delivered from March to June 2019. A midline survey was conducted 5–7 months (median 6 months) following intervention delivery from October to December 2019. The endline survey was originally planned to be conducted 6 months later. However, due to government lockdowns and restrictions from COVID-19, the endline survey was delayed by approximately 2 months and was completed 13–16 months (median 14 months) after intervention delivery from July-September 2020. We aimed to have equal number of intervention and control villages visited in a day. We collected drinking water samples and information on household and demographic characteristics, reported and observed WASH access based on the WHO/UNICEF Joint Monitoring Programme (JMP) core household survey questions^42^, reported and observed water treatment and handling practices, and caretaker-reported health of children under 5. Questions were directed to the primary cooks aged 18 and over. If the primary cook was unavailable or under 18, questions were directed to another household member aged 18 and over. Respondents were asked to confirm questions on individual children with their respective primary caregivers if they were available. Survey data were collected and managed using *REDCap* electronic data capture tools hosted at Emory University^43^.

### Primary and secondary outcomes

The primary outcome is detectable *E. coli* contamination of drinking water. Following the WHO/UNICEF JMP core household survey questions, each respondent was asked to serve drinking water. A 100 mL sample was collected at each follow-up visit in a sterile *Whirl-Pak®* bag containing sodium thiosulfate (Nasco, Madison, WI, USA) and kept on ice until tested within 8 h with *CompactDry™* (Nissui Pharmaceutical, Tokyo, Japan) media plates using membrane filtration procedures prescribed by UNICEF^44^. Samples were initially diluted to 50 mL in order to reduce the likelihood of plates that were too numerous to count (TNTC). If drinking water samples were visibly turbid, then they were subsequently diluted to 20 mL, 10 mL, and 5 mL based on the severity of turbidity. Plates were incubated at 30 degrees Celsius for 24 hours using an *IncuBox Thermocult* (Boehringer, Mannheim, Germany). One technician then counted and recorded individual *E. coli* CFU on each plate. Random spot checks were performed by managers to validate counts. Water quality results were double entered by two different staff. Plates that were TNTC were assigned a level of 300 CFUs. At least one duplicate and blank of distilled water were tested with samples daily. For duplicate samples, the results of both counts were summed and divided by the total volume processed. In order to obtain standardized totals per 100 mL, we normalized the CFU count by the total volume processed and multiplied the result by 100.

Secondary health outcomes include caregiver-reported diarrhea and healthcare visits for diarrhea within the previous 7-days in children under 5 years of age and under 2 years of age at follow-up visits. For reporting diarrhea in the previous 7-days, we followed the World Health Organization (WHO) standard definition, which defines diarrhea as three or more loose stools in a 24-hour period that can take the shape of a container^45^. For reporting healthcare visits, we asked caregivers if they sought medical care from a health clinic or CHW for any reported diarrhea cases within the previous 7-days following the WHO definition of diarrhea or caregiver’s interpretation of diarrhea. We also collected data on whether children had a toothache in the previous 7-days to serve as negative control to account for courtesy bias^46^.

We collected data on filter coverage, use, and acceptability at midline and endline visits. To measure filter coverage, we observed whether the household had the filter and if the filter was in good condition at the time of visit (e.g., assembled properly, working tap, no leaking, undamaged container, adequate flowrate, and ability to backwash). To measure filter use, we collected data on whether the filter was observed to have water in it at the time of visit and whether the household reported using the filter, filling the filter in the previous 7-days, treating drinking water, and if a child under 5 drank filtered water the previous day. To measure filter acceptability, we asked households to rate their acceptability of the appearance of filtered water, smell, taste of filtered water, and time to filter water on a scale from 1 to 4, with 3 and 4 being acceptable and very acceptable, respectively.

### Statistical approach

The study was powered to detect a 25% reduction in prevalence of detectable *E. coli* bacteria in point-of-use water samples, measured at each household visit. The number of households required in each group was derived by first using Diggle, Heagerty, Liang, and Zeger’s^47^ formula for estimating sample-size requirements for differences in proportions across multiple time points. The result of this equation was then adjusted to account for both village-level clustering and the assumed 15% rate of attrition. We assumed 50% prevalence of *E. coli* presence in drinking water samples in the control group based on national water quality surveys. We further assumed an intra-village correlation of 0.14 and intra-household ICC of 0.21 based on previous studies^18^, 2 visits postbaseline, and 25 households per village would meet eligibility requirements. This gave us a sample size requirement of 51 villages to have 80% power for a 25% reduction. To further accommodate the uncertainties of CHC enrollment rates and village size, we aimed to enroll up to 1300 households across 60 villages.

We defined the primary outcome as the presence of *E. coli* bacteria in 100 mL samples of drinking water. As the samples were diluted for purposes of this analysis, the presence of *E. coli* CFU follows the limit of detection (LOD) according to the volume processed. The laboratory results showed that the total volume of water processed for household samples that did not display any CFUs (e.g., non-detect plates) ranged from 50 mL to 100 mL. Therefore, results were categorized into a binary variable, where non-detectable *E. coli* contamination is overall reported as <2 CFU/100 mL water (e.g., LOD for a 50 mL sample). We additionally categorized *E. coli* presence into two other binary outcomes according to WHO risk category cutoffs for moderate-to-high (≥10 CFU/100 mL) and very high (≥100 CFU/100 mL) contamination^20^. We examined the latter outcomes based on findings from meta-analysis on water quality and diarrhea, which found a marked increase in disease risk for households when fecal contamination exceeded 10 TTC/100 mL^33^. We calculated arithmetic and Williams means of CFU counts to account for the skewed distribution. The Williams mean is calculated by adding 1 to all values, taking the geometric mean, and then subtracting the mean by 1^48^. Williams mean were used to account for values less than 1. Non-detect plates were included in the mean calculation as half of their specific LOD.

The effect of the intervention was assessed based on group assignment, regardless of uptake of the intervention (i.e. intention-to-treat). For the household-level primary outcomes on *E. coli* presence in drinking water and the individual-level secondary outcomes on child health, we used binomial regression with a log link and generalized estimating equations (GEE) with robust standard errors to account for village-level clustering^49,50^. For the child health models, we adjusted for sex and age in months. We estimated prevalence ratios by calculating the exponential of the model coefficients for the group assignment. Statistically significant effect of the assignment were determined by using a two-sided Type I error rate of 0.05. We provide sample proportions and 95% confidence intervals for outcomes on filter coverage, acceptability, and use in the intervention group.

#### Covariate adjustment for imbalance

We reviewed the baseline data to see if there were large differences (>10% difference) between arms in socio-economic and household variables that are established determinants of drinking water quality or childhood diarrhoea (Table 1). Covariates that had little variation in the study population (e.g., over 95% prevalence or less than 5% prevalence) were excluded from adjustment. We then examined the relationship between primary and secondary outcomes and imbalanced covariates of concern (e.g., socioeconomic status, access to handwashing location, and access to improved sanitation) in individual bivariate analyses. Socioeconomic status was associated with diarrheal prevalence in children under 5 and 2 (*p* < 0.05). Access to a handwashing location was associated (*p* < 0.05) with only very high levels of *E. coli* bacteria (≥100 CFU/100 mL) and to diarrheal prevalence in children under 5 (*p* < 0.05). Access to sanitation was not associated with any outcome. We adjusted for socio-economic status and access to handwashing station in separate sensitivity analyses and compared results to unadjusted models to see if there were considerable differences in effects of the intervention. Water quality effects observed in unadjusted models were comparable to models adjusted for access to handwashing. Effects on under-5 child diarrhoea prevalence from the intervention had a 5 percent difference between the unadjusted model and adjusted model with socioeconomic status. Effects on under-5 child diarrhoea prevalence from the intervention had less than one percent difference between the unadjusted model and adjusted model with access to handwashing location. Therefore, we chose to only adjust for socio-economic status in all final models. Unadjusted and adjusted models are presented together in Tables 3 and 4.

#### Clustering considerations

Current GEE statistical packages are limited in that they only allow for adjusting for one level of clustering. We adjusted at the village-level because it is the highest level of clustering that is of concern and the unit of randomization^51^, which should intrinsically adjust for lower levels of clustering^49^. In sensitivity analyses, we adjusted for household-level clustering to account for longitudinal sampling, but did not see major differences in the water quality or diarrhea effects compared to the models adjusted for village-level clustering. The comparison in presented in the water quality results in Supplementary Table 2.

All analyses were done using Stata 16 (Stata Corporation, College station, TX, USA)^52^.

### Ethics and registration

The trial is registered under the Pan African Clinical Trial Registry, Trial ID = PACTR201812547047839. The protocol received ethical approval and was annually renewed by the Emory University Institutional Review Board (CR001-IRB00106424) and Rwanda National Ethics Committee (IRB 0001497). We obtained signed informed consent from the main survey respondent during enrollment.

## Supplementary information

Supplementary infromation

## Data Availability

Study protocol and underlying de-identified data can be found at Emory/UNC Dataverse. Users must provide a description of how data will be used before downloading datasets. 10.15139/S3/H3UJMQ.

## Code availability

Corresponding analysis code central to the conclusions of this study are available from the authors upon request.
