# Supplementary material for: Effects of adding household water filters to Rwanda’s Community-Based Environmental Health Promotion Programme: a cluster-randomized controlled trial in Rwamagana district
Source: NPJ Clean Water. 2022 Sep 12;5(1):42. doi: 10.1038/s41545-022-00185-y (PMC9464616; doi:10.1038/s41545-022-00185-y)
Supplement: Supplementary file 1 — Supplementary infromation [file 41545_2022_185_MOESM1_ESM.pdf]

Supplementary Information for

Effects of adding household water filters to Rwanda's Community-Based Environmental Health Promotion Programme: A cluster-randomized controlled trial in Rwamagana district

Sabrina Haque, Miles A. Kirby, Laurien Iyakaremye, Alemayehu Gebremariam, Getachew Tessema, Evan Thomas, Howard H. Chang, and Thomas Clasen*

*Corresponding author
thomas.f.clasen@emory.edu
1518 Clifton Road NE, Atlanta GA 30322

Contents

1. Supplementary Table 1. Means of CFU count per 100 mL of drinking water samples by study group and round
2. Supplementary Table 2. Sensitivity analyses - effects of intervention during follow-up on household-level drinking water quality outcomes adjusting for repeated household measurements

Supplementary Table 1. Means of CFU count per 100 mL of drinking water samples by study group and round

	Intervention			Control		
**Visit**	**N**	**AM (95% CI)**	**WM (95% CI)**	**N**	**AM (95% CI)**	**WM (95% CI)**
Midline	448	88.6 (73.0, 104.2)	14.9 (12.3, 18.0)	366	174.5 (148.1, 200.8)	43.6 (35.6, 53.3)
Endline	481	94.8 (78.7, 111.0)	13.4 (11.0, 16.2)	473	175.9 (153.8, 198.0)	45.0 (37.5, 54.0)
Overall	929	91.8 (80.6, 103.1)	14.1 (12.3, 16.1)	839	175.3 (158.3, 192.2)	44.4 (38.8, 50.8)

AM: Arithmetic mean; WM: Williams Mean. To calculate WM, 1 was added to the variable before taking the geometric mean to account for values less than 1 and then the result was subtracted by 1.

Supplementary Table 2. Sensitivity analyses - effects of intervention during follow-up on household-level drinking water quality outcomes adjusting for repeated household measurements

Model	Drinking water quality	Intervention	Control	PR (95% CI)	*p*
1[1]	≥2 CFU/100 mL (any detectable *E.coli contamination*)	69.9% (649/929)	87.0% (730/839)	0.80 (0.76, 0.85)	<0.001
2[1]	≥10 CFU/100 mL (Moderate and higher *E.coli* contamination)	49.3% (458/929)	74.7% (627/839)	0.66 (0.61, 0.72)	<0.001
3[1]	≥100 CFU /100 mL (Very high *E.coli contamination*)	22.4% (208/929)	39.8% (334/839)	0.56 (0.48, 0.66)	<0.001
4[2]	≥2 CFU/100 mL (any detectable *E.coli contamination*)	69.8% (644/923)	87.2% (728/835)	0.80 (0.76, 0.84)	<0.001
5[2]	≥10 CFU/100 mL (Moderate and higher *E.coli* contamination)	49.2% (454/923)	75.0% (636/835)	0.66 (0.61, 0.71)	<0.001
6[2]	≥100 CFU /100 mL (Very high *E.coli contamination*)	22.4% (207/923)	39.8% (333/835)	0.56 (0.48, 0.65)	<0.001

n denotes the total number of household water samples analyzed in follow-up rounds.

[1] Prevalence ratio (PR), 95% Confidence Interval (95% CI) and p-value derived from log-binomial generalized estimating equations with robust standard errors to account for clustering within household from repeated measurements. Model only conditions group assignment and drinking water quality outcome.

[2] PR, 95% CI and p-value derived from log-binomial generalized estimating equations with robust standard errors to account for clustering within household from repeated measurements. Model further adjusts for government-defined socio-economic status.
